# Stevens-Johnson Syndrome in a Patient With Recent SARS-CoV-2 Infection and Ciprofloxacin Administration

**DOI:** 10.7759/cureus.45099

**Published:** 2023-09-12

**Authors:** Evgenia Skafida, Rafail Giannas

**Affiliations:** 1 Internal Medicine, General Hospital of Syros, Ermoupoli, GRC

**Keywords:** sars-cov-2, ciprofloxacin, corticosteroid treatment, toxic epidermolysis necrosis, stevens-johnson syndrome (sjs)

## Abstract

Stevens-Johnson syndrome (SJS) is an acute, rare, and potentially life-threatening condition with high morbidity and mortality. It is characterized by a blistering rash and erosions with mucosal involvement, which depending on the extent of the skin area involved may be categorized as epidermal necrolysis, along with systemic symptoms. Symptoms are preceded by the administration of a newly introduced drug in almost 80% of cases and less commonly by infections in genetically predisposed individuals. We report a case of SJS in a female patient secondary to a recent SARS-CoV-2 infection and subsequent ciprofloxacin administration.

## Introduction

Stevens-Johnson syndrome (SJS) is a rare entity. The syndrome can be subdivided into SJS in cases of involvement of body surface area (BSA) below 10%, SJS/toxic epidermal necrolysis (TEN) overlap in 10-30% BSA, or TEN when skin detachment extends beyond 30% BSA [1]. SJS can be fatal, as mortality might reach 90% of cases. The presence of an underlying malignancy, older age, tachycardia, increased serum urea or hyperglycemia, or initial BSA involvement over 10% are bad prognostic factors [1]. SJS has been noted to primarily affect women and the elderly [2].

SJS can be expressed days or even weeks after exposure to a potential trigger, which most frequently is a pharmaceutical substance. In most cases, the syndrome is a result of antiepileptic drug use such as carbamazepine, but it can also be brought about by other medicaments, for example, allopurinol, non-steroidal anti-inflammatory drugs, and sulfonamides [3]. More rarely, infections can be at fault. Such examples can be cytomegalovirus and mycoplasma infections [2]. Several cases of SJS secondary to an infection with SARS-CoV-2 have also been recorded [4].

## Case presentation

A 73-year-old female patient came to our emergency department due to malaise and a painful bullous eruption affecting her thorax and upper and lower extremities and also involving the mucosal membranes of her mouth and anus, which developed five days after initiating a ciprofloxacin course. The antibiotic was prescribed to her by a pulmonologist due to a COVID-19 infection that was confirmed two weeks before her presentation to the emergency room. She initially visited a dermatologist because of the eruption, who advised her on a course of miconazole oral gel, methylprednisolone 16mg daily, and levocetirizine 5mg daily, with further deterioration of her clinical picture. 

From her medical history, arterial hypertension and dyslipidemia were noted. She was being treated with amlodipine 5mg, flecainide 100mg, rosuvastatin 20mg, metoprolol 25mg, ramipril 5mg, and salicylic acid 100mg once daily. The patient was triple vaccinated with Comirnaty and her last vaccination was administered about six months prior to the current presentation. 

**Figure 1:**
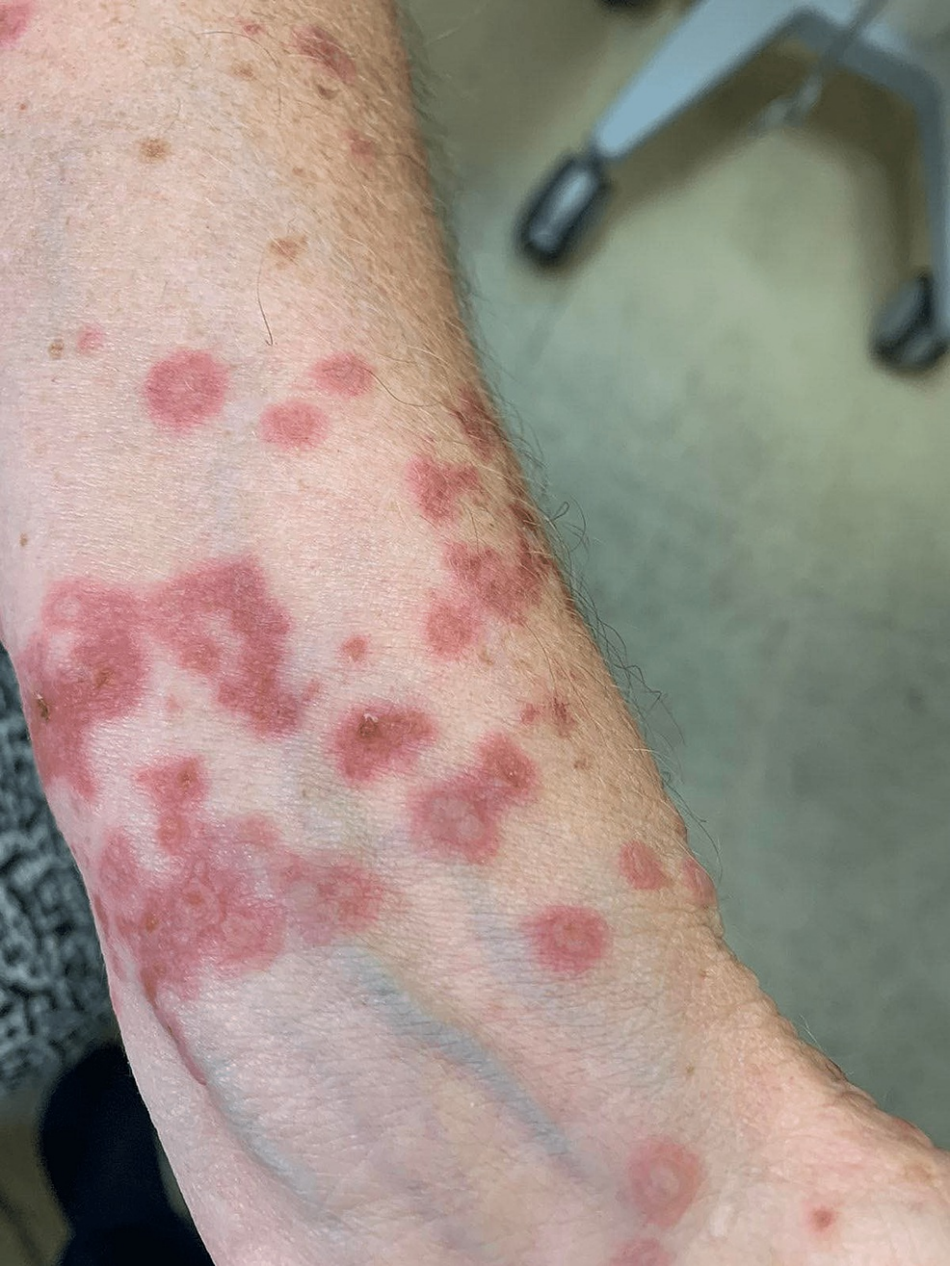
Patient's left wrist upon presentation

**Figure 2:**
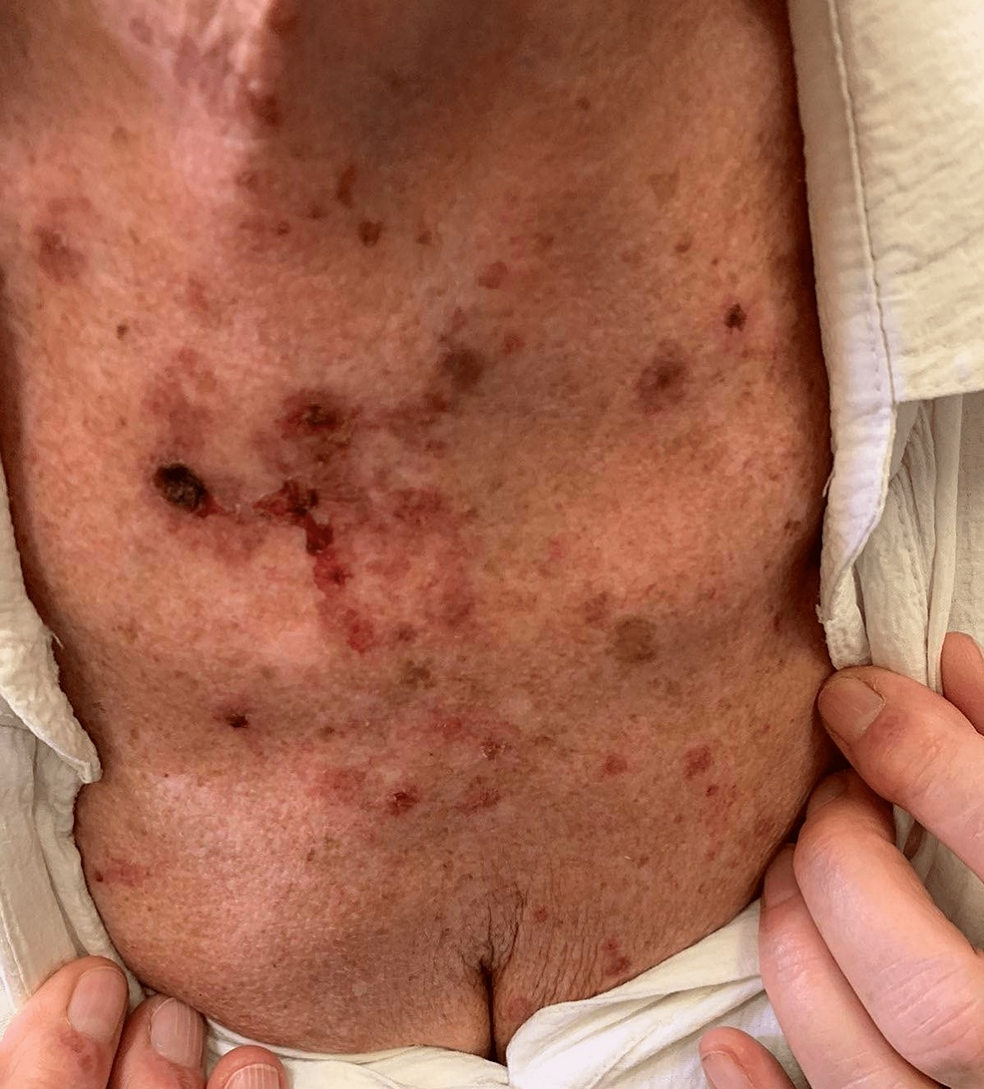
Chest of the patient upon presentation showing erythematous macules, bullae, and hemorrhagic crusts

**Figure 3:**
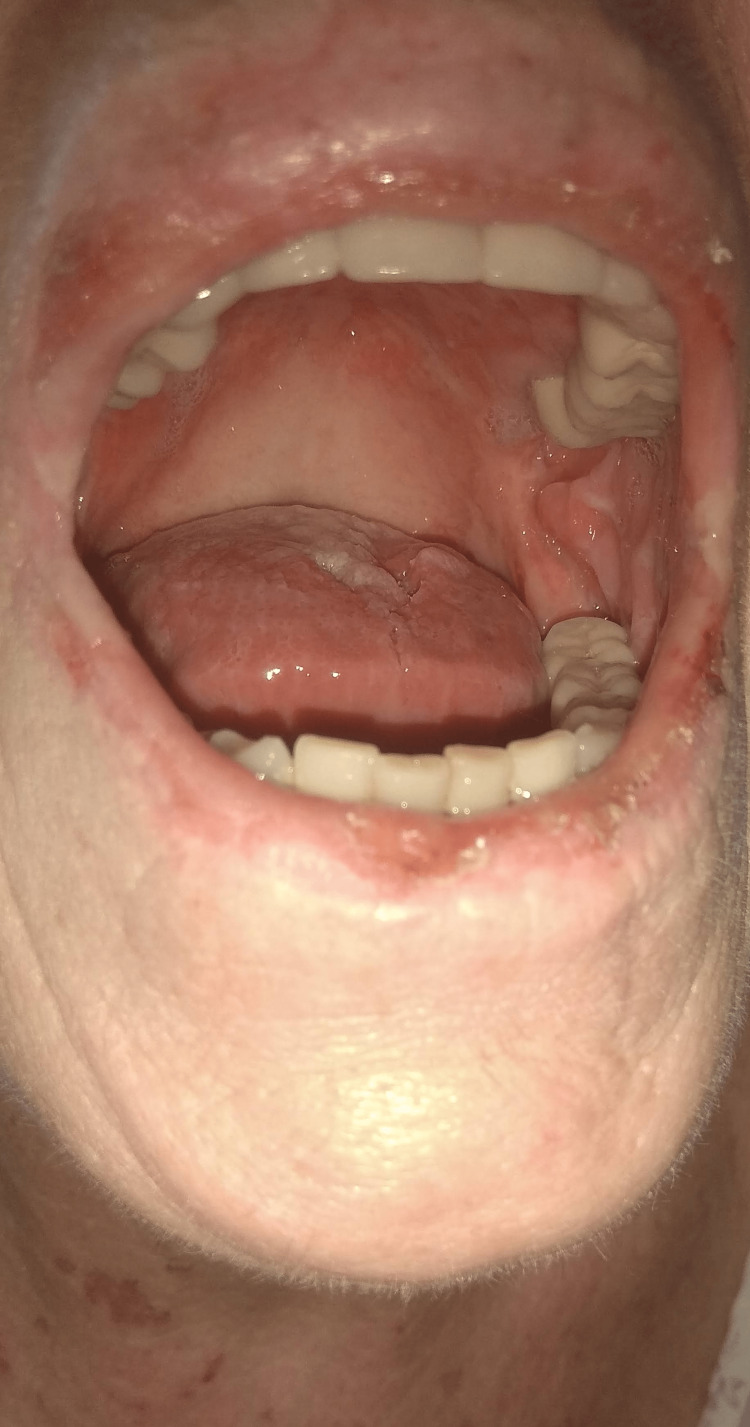
Mouth of the patient. The image was obtained after the initiation of treatment

Upon presentation, the patient appeared fatigued. Her blood pressure was 117/67 mmHg, she had a heart rate of 86 beats per minute, a temperature of 36.3°C, and an oxygen saturation of 95% on ambient air. An erythematous rash and blisters were noted on her trunk and upper and lower extremities bilaterally (Figures 1-3). The mucosal membranes of her mouth, nasal cavity, and anus were affected as well. The diagnosis was confirmed by a dermatology specialist, who did not request a skin biopsy. The extent of the lesions was estimated to cover a BSA of about 9%; all findings suggesting the diagnosis of SJS. 

Lab tests showed an ESR of 28 (NL 0-20), CRP 32,8 (NL <5), WBC 17150 (NL 4000-11000), and neutrophils 86% (NL 30-70%). No further pathological laboratory findings were noted, including hepatic and renal function tests. CEA, CA19-9, and CA-125 tumor markers turned up negative. Rapid antigen testing for COVID-19 as well as PCR testing was negative upon her presentation.

**Figure 4:**
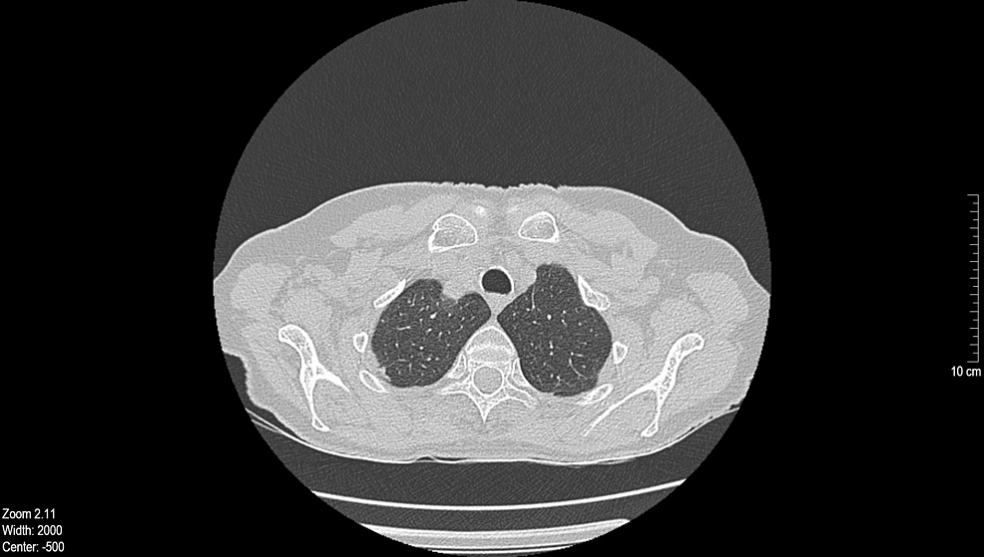
Chest CT of the patient showing a subpleural nodule

Chest CT scan revealed subpleural nodules of about 3mm diameter in both upper lobes of the lungs as well as linear fibrous elements in the lingula and both lung bases, likely of a post-inflammatory etiology, as well as areas with bronchiectasis (Figure 4). 

The patient was admitted to the internal medicine ward. After dermatologic consultation and confirmation of the diagnosis, she was put on a methylprednisolone 500mg intravenous regimen for three days, a proton pump inhibitor, and levocetirizine 5mg twice a day. Flecainide and metoprolol were continued, and the rest of her regimen was temporarily discontinued. She remained hemodynamically stable with a slightly improved clinical picture. On this basis, she was transferred to the dermatologic clinic of a tertiary hospital, where the presumptive diagnosis of SJS was accepted and she was further treated accordingly. Subsequently, the patient was discharged on a de-escalating oral prednisolone regimen.

## Discussion

SJS and TEN are diagnoses with no universally accepted criteria. Histopathologic findings are neither diagnostic nor specific. The diagnosis of SJS is suggested when there is a history of drug exposure especially to drugs that have previously been associated with the syndrome in a patient with malaise and/or a prodromal febrile illness. SJS and TEN are also characterized by the presence of a painful, symmetric rash consisting of erythematous macules, targetoid lesions, or diffuse erythema rapidly progressing to vesicles and bullae, which usually first appear on the face and trunk. The epidermis demonstrates sloughing and necrosis. Painful oral, ocular, and genital lesions are also suggestive of the diagnosis. They usually affect women as well as the elderly and are immunocompromised [1].

The Naranjo scale of adverse drug reaction probability takes into account 10 parameters including previously documented similar reactions to a specific drug, previous exposure of the patient to a given drug, time of onset of reaction in relation to the administration of the drug, and alternative causes of the reaction [5]. SJS in this patient appears to have occurred secondary to ciprofloxacin use. It is concluded that as there are reported cases of SJS after ciprofloxacin administration [6-8], symptoms were first noted five days after starting the regimen and improved upon withdrawal and no previous exposure to this particular antibiotic was documented. Also, no placebo was administered, the patient was not known to have previously taken any similar antibiotics, and the dosage of ciprofloxacin was not modified.

In addition, our patient’s regimen contained medicaments that could potentially trigger SJS. Those can safely be excluded as a potential cause, as they were well tolerated for an extended amount of time. Also, no adjustment was done to her regular regimen, nor did she receive any other medications or supplements at any point in time. 

As a conclusion, in the above-mentioned case there is an exposure to a drug that is known to potentially trigger SJS. However, based on the Algorithm of Drug Causality in Epidermal Necrolysis (ALDEN) (Table 1) [9] there is a weaker association between the development of SJS and ciprofloxacin administration as compared to some other medications (Table 2). However, given the available bibliography [4], SJS can also be triggered by SARS-CoV-2 infection. To be more precise, cases were described where SJS was confirmed up to 42 days after SARS-CoV-2 infection [4], while in this patient the symptoms of SJS appeared within about two weeks post-infection. 

**Table 1:** ALDEN algorithm applied for this case (in bold) giving ciprofloxacin a score of 5 and thus a probable causality with SJS <0, Very unlikely; 0–1, unlikely; 2–3, possible; 4–5, probable; ≥6, very probable. ATC, anatomical therapeutic chemical; SJS, Stevens–Johnson syndrome; TEN, toxic epidermal necrolysis; ALDEN: Algorithm of Drug Causality in Epidermal Necrolysis ^a^Drug (or active metabolite) elimination half-life from serum and/or tissues (according to pharmacology textbooks), taking into account kidney function for drugs predominantly cleared by kidney and liver function for those with high hepatic clearance. ^b^Suspected interaction was considered when more than five drugs were present in a patient’s body at the same time. ^c^Similar drug = same ATC code up to the fourth level (chemical subgroups).

Criterion	Values	Rules to Apply
Delay from initial drug component intake to onset of reaction (index day)	Suggestive +3	From 5 to 28 days
	Compatible +2	From 29 to 56 days
	Likely +1	From 1 to 4 days
	Unlikely −1	>56 Days
	Excluded −3	Drug started on or after the index day
		In case of previous reaction to the same drug, only changes for: Suggestive: +3: from 1 to 4 days Likely: +1: from 5 to 56 days
Drug present in the body on index day	Definite 0	Drug continued up to index day or stopped at a time point less than five times the elimination half-life^a^ before the index day
	Doubtful −1	Drug stopped at a time point prior to the index day by more than five times the elimination half-life^a^ but liver or kidney function alterations or suspected drug interactions^b^ are present
	Excluded −3	Drug stopped at a time point prior to the index day by more than five times the elimination half-life^a^, without liver or kidney function alterations or suspected drug interactions^b^
Prechallenge/ rechallenge	Positive specific for disease and drug: 4	SJS/TEN after use of same drug
	Positive specific for disease or drug: 2	SJS/TEN after use of similar drug^c^ or other reaction with same drug
	Positive unspecific: 1	Other reaction after use of similar drug^c^
	Not done/unknown: 0	No known previous exposure to this drug
	Negative −2	Exposure to this drug without any reaction (before or after reaction)
Dechallenge	Neutral 0	Drug stopped (or unknown)
	Negative −2	Drug continued without harm
Type of drug (notoriety)	Strongly associated 3	Drug of the “high-risk” list according to previous case–control studies
	Associated 2	Drug with definite but lower risk according to previous case–control studies
	Suspected 1	Several previous reports, ambiguous epidemiology results (drug “under surveillance”)
	Unknown 0	All other drugs including newly released ones
	Not suspected −1	No evidence of association from previous epidemiology study with sufficient number of exposed controls
		Intermediate score = total of all previous criteria
Other cause	Possible −1	Rank all drugs from highest to lowest intermediate score
		If at least one has an intermediate score >3, subtract 1 point from the score of each of the other drugs taken by the patient (another cause is more likely)
Final score −12 to 10		

**Table 2:** Examples of medications associated with SJS and their ALDEN scores ALDEN: Algorithm of Drug Causality in Epidermal Necrolysis

Drug name	ALDEN score
Sulfasalazine	6
Etoricoxib	5,75
Lamotrigine	5.67
Allopurinol	5.18
Carbamazepine	5
Phenytoin	4.5
Cefuroxime	4
Amoxicillin – Clavulanic acid	3.25
Ciprofloxacin	3
Amoxicillin	3

## Conclusions

We presented a case of SJS with possible trigger either by the use of ciprofloxacin or the recent infection of the patient by SARS-CoV-2. Ciprofloxacin is a rare but established cause of SJS with an ALDEN score of 3. Recent publications point out a possible connection between infection or vaccination against SARS-CoV-2 and SJS. This case is of note because of the likelihood that SJS may have been triggered by the recent SARS-CoV-2 infection.
